# Coronary artery bypass grafting in Jehovah’s Witness patients: a retrospective propensity matched cohort study

**DOI:** 10.1016/j.bjane.2026.844765

**Published:** 2026-05-13

**Authors:** Henrique Ryu Yamanaka Nakano, Guilherme Mota Filho, Bruno Camargo Calabria Araújo, Heloísa Zogheib, Enzo Camargo Calabria Araújo, Leonardo Cardoso Saraiva, Jessica Silva Nicolau, Suely Pereira Zeferino, José Otávio Costa Auler Junior

**Affiliations:** a Hospital de Clínicas da Faculdade de Medicina da Universidade de São Paulo, Anesthesiology Institute of Surgery Department, São Paulo, SP, Brazil; b Hospital de Clínicas da Faculdade de Medicina da Universidade de São Paulo, Heart Institute, São Paulo, SP, Brazil

**Keywords:** Blood component transfusion, Coronary artery bypass, Jehovah’s Witnesses, Perioperative care, Transfusion-free surgery

## Abstract

**Background:**

Coronary Artery Bypass Grafting (CABG) in Jehovah’s Witness (JW) patients raises concerns regarding perioperative safety because of refusal of allogeneic blood transfusions. This study evaluated the feasibility and short-term outcomes of CABG in JW patients and described perioperative blood management practices.

**Methods:**

This retrospective observational study included 26 JW patients and 78 matched non-Jehovah’s Witness (non-JW) patients who underwent CABG at InCor HC-FMUSP between 2015 and 2023. Propensity score matching at a 3:1 ratio based on age, sex, and preoperative hemoglobin was used to improve comparability. The primary outcome was 30-day all-cause mortality. Secondary outcomes included 30-day stroke, myocardial infarction, and acute kidney injury; ICU length of stay; perioperative red blood cell transfusion rates; postoperative hemoglobin and hematocrit; intraoperative blood loss; and cardiopulmonary bypass time.

**Results:**

No 30-day deaths occurred among JW patients, whereas three deaths occurred in the non-JW group (OR = 0.45; 95% CI 0.003–5.09; p = 0.575). No JW patient experienced stroke, and one myocardial infarction occurred. JW patients had higher postoperative hemoglobin (OR = 1.50; 95% CI 1.03–2.28; p = 0.032) and lower intraoperative blood loss (OR = 0.55; 95% CI 0.27–0.98; p = 0.046). No JW patient received transfusion, whereas 17.9% and 12.8% of non-JW patients required intraoperative and postoperative transfusions, respectively. ICU length of stay was similar between groups.

**Conclusion:**

In this cohort, CABG in JW patients was feasible with acceptable short-term outcomes. Outcomes were generally comparable between groups, acknowledging imprecision related to the small JW sample.

## Introduction

Advances in cardiac surgery have substantially improved survival and quality of life among patients undergoing coronary artery bypass grafting (CABG).^1^ Despite these advances, clinical management may be influenced by patient-specific factors, including religious beliefs that restrict certain therapeutic options. Jehovah’s Witnesses (JW), for example, refuse allogeneic blood transfusions for religious reasons, a decision that can pose complex clinical and ethical challenges in procedures associated with significant bleeding risk.^2^^,^^3^ In the context of CABG, this refusal has traditionally raised concerns regarding perioperative safety and, in some settings, has led to delays or even denial of surgical treatment.^4^, ^5^, ^6^

Although prior studies have reported acceptable CABG outcomes in JW patients, most originate from selected populations or high-resource centers. Data from public healthcare systems remain limited, particularly in contexts characterized by prolonged waiting times and variable access to blood conservation technologies. Our findings contribute to evidence from such a setting.

Blood loss during CABG is a major determinant of perioperative morbidity and mortality.^7^ While transfusion practices were historically liberal, contemporary evidence supports restrictive strategies in cardiac surgery, emphasizing meticulous hemostasis, judicious blood product use, and structured perioperative care.^8^^,^^9^ For patients who refuse transfusion, these principles are essential and require coordinated blood conservation approaches, including preoperative anemia management, intraoperative cell salvage, and pharmacologic optimization.

This study evaluated perioperative management, implementation of Patient Blood Management (PBM) strategies, and short-term outcomes of JW patients undergoing CABG in a contemporary public hospital using a retrospective, propensity-matched observational design, with focus on 30-day morbidity and mortality.

## Methods

### Study design

This was a single-center, retrospective observational cohort study comparing JW and non-JW patients undergoing isolated CABG. This manuscript was prepared in accordance with the Strengthening the Reporting of Observational Studies in Epidemiology (STROBE) guideline. A completed STROBE checklist is provided as supplementary material.

### Ethics approval and consent

All JW patients underwent individualized preoperative informed consent, during which acceptable and non-acceptable blood products and hemostatic agents were discussed and documented in the medical record, in accordance with patient autonomy and institutional ethical standards. Owing to the retrospective design, no standardized registry allowed systematic determination of how many patients explicitly consented to transfusion in life-threatening situations, although such discussions were part of routine care.

This retrospective observational study used anonymized medical record data without direct patient contact. The requirement for informed consent was waived by the Ethics Committee (Approval n° 7.441.177; CAAE 85677324.8.0000.0068; March 14, 2025).

### Participants

We included 26 JW and 78 matched non-JW patients who underwent isolated CABG at the Heart Institute, University of São Paulo, between January 2015 and December 2023.

### Inclusion and exclusion criteria

Adults (> 18 years) undergoing first-time isolated CABG were included. Patients undergoing reoperation or combined procedures (e.g., valve or aortic surgery) were excluded.

### Patient selection

**Figure 1:**
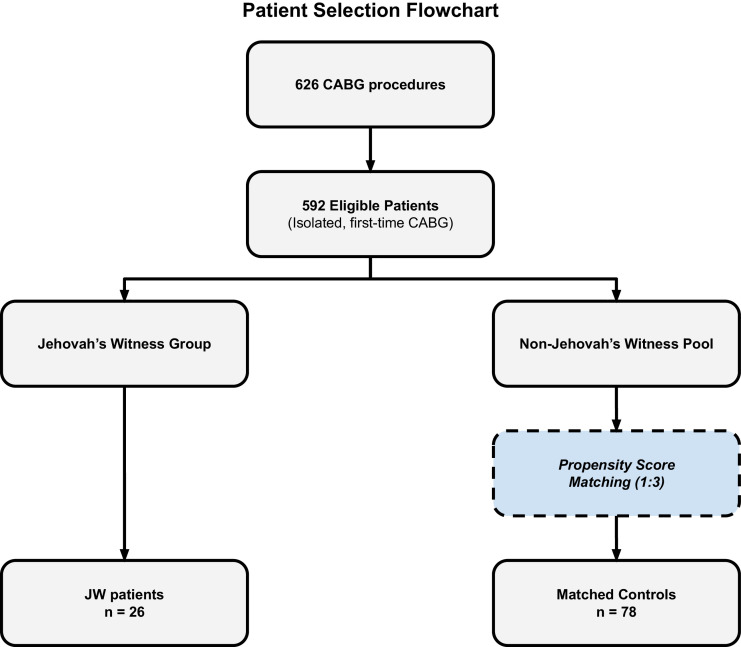
Flow diagram of patient selection and matching CABG, Coronary artery bypass grafting. Patients excluded from the initial screening (n=34) consisted of reoperations or combined procedures. Propensity score matching was performed using a 1:3 ratio to ensure balanced baseline characteristics.

The JW cohort (n = 26) comprised all consecutive JW patients undergoing isolated CABG during the study period. No a priori sample size calculation was performed, as this represented the total eligible JW population. Controls were selected in a 1:3 ratio from 566 non-JW patients who underwent isolated CABG by the same surgeon during the same period. As this surgeon is the only operator performing cardiac surgery in JW patients at our institution, restricting controls to his case list was intended to reduce surgeon-related variability and improve comparability before propensity score matching. The screening and matching process is summarized in the Figure 1 below.

### Statistical analysis

**Figure 2:**
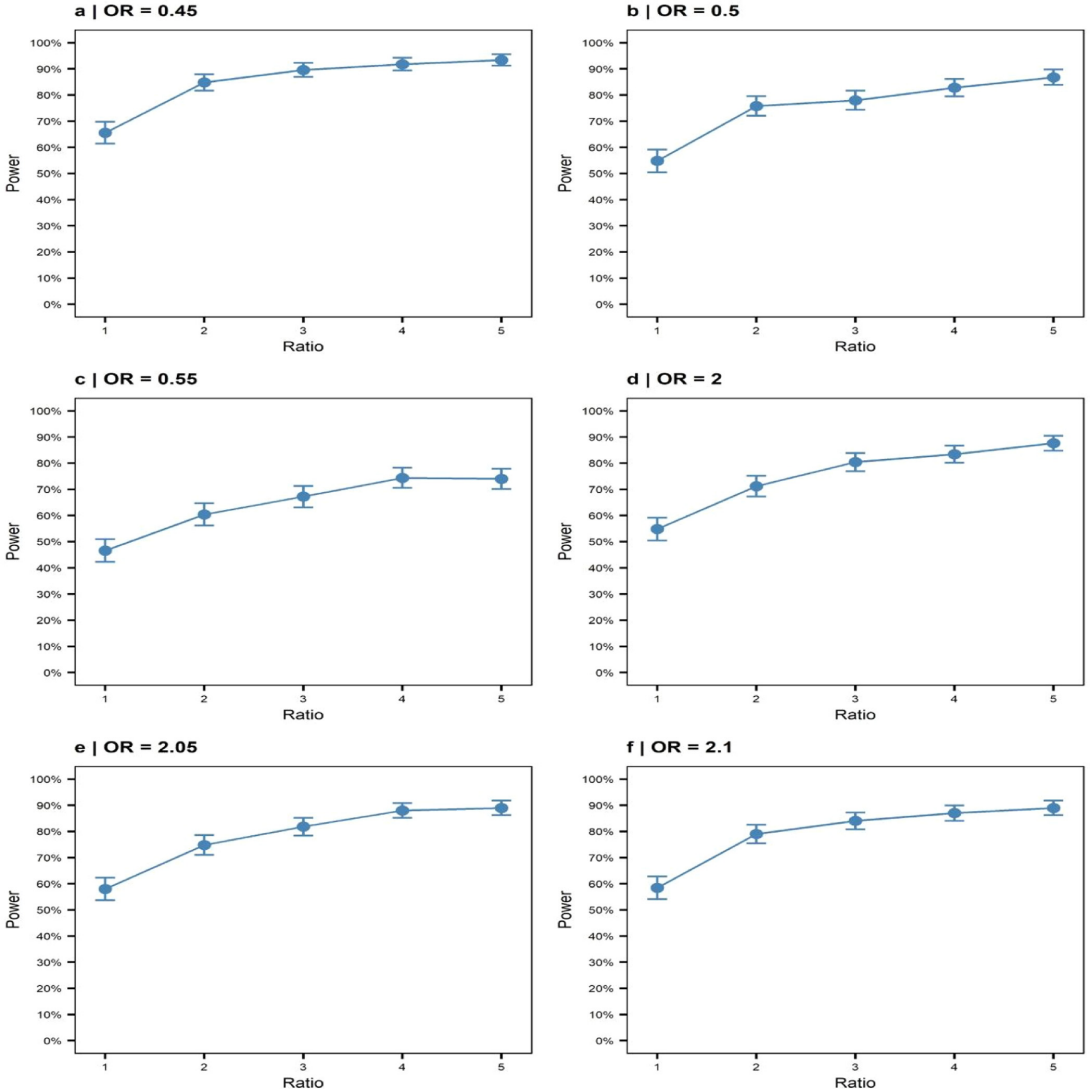
Simulation-based power analysis for defining the best control-to-case ratio to be used in propensity score matching. Each panel shows power estimates (±95% CI) as a function of the control-to-case ratio (x-axis), assuming a fixed number of cases (n=26) and a corresponding fixed number of controls (n = ratio × 26). Panels (a–c) show scenarios with OR < 1 (protective effects), and panels (d–f) show OR > 1 (risk effects). Each curve is based on 500 simulations. Power increases with larger control-to-case ratios, with diminishing gains beyond a 1:3 ratio.

To address the sample size disparity between JW and non-JW patients, Propensity Score Matching (PSM) was performed using age, sex, and preoperative hemoglobin.^10^^,^^11^ We used nearest-neighbor matching, without replacement and without a caliper restriction. The case-to-control ratio was selected via a simulation-based power analysis evaluating ratios from 1:1 to 5:1.^12^ In each simulation, a continuous predictor and a binary outcome with fixed log-odds were generated using 26 cases and varying control ratios. Firth-penalized logistic regression was fit in 500 simulations per scenario, with power defined as the proportion of p-values < 0.05.^13^ As shown in Figure 2, power plateaued at a 3:1 ratio, which was adopted.

Baseline demographic and clinical characteristics were compared between JW and non-JW patients using Chi-square tests for categorical variables and *t*-tests or Mann-Whitney *U*-tests for normal and non-normal continuous variables, respectively. Logistic regression compared groups across pre-, intra-, and postoperative outcomes. More specifically, in each model, group was modeled as the dependent variable and the respective clinical outcome as the independent variable. Models were additionally adjusted for age, sex, and preoperative hemoglobin to account for residual imbalance after matching.^14^ Firth's correction was used due to binary outcomes with rare events (including zero counts) like transfusion and death.^13^ The Shapiro-Wilk test assessed normality of continuous variables; non-normal variables were log-transformed. Odds Ratios (ORs) were calculated by exponentiating model coefficients, with 95% Confidence Intervals estimated via profile likelihood; p-values were not adjusted for multiple comparisons to preserve statistical power.

We performed a sensitivity analysis to assess the potential impact of clinical characteristics on the assessed outcomes. This was done by including all of the available clinical characteristics (EuroSCORE II, ASA status, LVEF < 50%, diabetes, and hypertension) in the logistic regression models and re-running them. Given its role as a validated composite predictor of surgical mortality and morbidity in CABG, adjustment for EuroSCORE II was considered the main analytic framework for outcome estimation. This approach was selected to provide clinically meaningful risk adjustment beyond the matching variables while preserving model stability in a small sample.

Because of the small number of JW patients and the risk of model overparameterization, the propensity score was restricted to key baseline variables directly associated with transfusion exposure and perioperative risk. EuroSCORE II, as a composite risk index, was included in regression adjustment models rather than in the matching algorithm to maintain balance and model stability.

There were no missing data for variables included in the analysis. All analyses were conducted using R (R Core Team, 2025) using the MatchIt^15^ and Logistf packages.^16^

### Predictors

#### Preoperative predictors

Included age, sex, American Society of Anesthesiologists (ASA) status (1–5), EuroSCORE II, preoperative Hemoglobin (Hb) and Hematocrit (Ht) measured the day before surgery, and comorbidities (diabetes mellitus, hypertension, dyslipidemia, Coronary Artery Disease [CAD], and heart failure with Left Ventricular Ejection Fraction [LVEF] < 50%). Reported Hb values reflect measurements obtained immediately before surgery and therefore incorporate any preoperative optimization, including iron or erythropoietin when used. Diabetes, hypertension, and dyslipidemia were defined by prior diagnosis or ongoing pharmacologic treatment. CAD was defined as prior myocardial infarction, coronary revascularization, or angiographically confirmed disease. Left ventricular dysfunction was defined as LVEF < 50%.

#### Perioperative blood management

Preoperative anemia optimization differed between groups. In JW patients, iron and erythropoietin were used selectively based on Hb levels, surgical urgency, and patient consent. When erythropoietin was administered, surgery was performed after achieving clinically acceptable Hb levels (typically ≥ 12.0 g.dL^-1^), balancing optimization with urgency. In non-JW patients, anemia management followed routine institutional practice without a standardized protocol. Tranexamic acid was routinely administered intraoperatively according to institutional CABG protocols. No additional hemostatic agents were systematically used preoperatively in either group.

#### Transfusion policy

In the non-JW group, transfusion decisions were based on multifactorial clinical assessment rather than a fixed Hb threshold, incorporating active bleeding, hemodynamic instability, tissue perfusion, and overall clinical context. In this study, “transfusion” refers exclusively to allogeneic Red Blood Cell (RBC) transfusion; use of plasma, platelets, or other hemostatic agents (e.g., fibrinogen or prothrombin complex concentrate) was not analyzed, representing a limitation of the study. No JW patient required transfusion. Per institutional policy, management prioritized blood conservation and avoidance of transfusion in accordance with documented patient preferences. A contingency plan allowed RBC transfusion in life-threatening situations with imminent risk of death after multidisciplinary discussion and ethical review; no such cases occurred.

### Intraoperative predictors

Included Cardiopulmonary Bypass (CPB) duration (< 60, 60–120, > 120 minutes), estimated blood loss, and intraoperative transfusion. All JW patients had access to intraoperative cell salvage. Among non-JW controls, cell-saver use was available in 43 of 78 cases, reflecting institutional resource availability rather than clinical selection. Perioperative anticoagulant and antiplatelet management followed institutional CABG protocols, including preoperative discontinuation and postoperative resumption according to bleeding and thrombotic risk. Blood loss was estimated by sponge weight and suction volume, a commonly used method with recognized limitations, including estimation error, occult loss, and dilution by irrigation fluids.

### Outcomes

The primary outcome was 30-day all-cause mortality. Secondary outcomes included 30-day stroke, Myocardial Infarction (MI), and Acute Kidney Injury (AKI); duration of mechanical ventilation; Intensive Care Unit (ICU) length of stay; perioperative Red Blood Cell (RBC) transfusion; use of vasoactive drugs; postoperative hemoglobin and hematocrit; intraoperative blood loss; and Cardiopulmonary Bypass (CPB) time.

The first hemoglobin and hematocrit measurement after ICU admission was used as the postoperative reference to reduce confounding from repeated sampling or non-surgical blood loss, acknowledging potential volemic shifts. Death was defined as intraoperative or all-cause mortality within 30 days. Vasopressor use included norepinephrine, epinephrine, or vasopressin; inotropes included dobutamine, epinephrine, or milrinone. Postoperative vasodilators (e.g., nitroprusside, nitroglycerin, hydralazine) were used for blood pressure control.

Peri-procedural MI was defined according to contemporary CABG criteria based on biomarker elevation with electrocardiography or imaging evidence. Stroke was defined as a new neurological deficit lasting > 24 hours, confirmed by neuroimaging after clinical suspicion. AKI was defined per Kidney Disease: Improving Global Outcomes (KDIGO) criteria, using baseline creatinine measured within 48 hours preoperatively and assessed during the first 7 postoperative days or until discharge.

## Results

### Demographic characteristics (Table 1)

Baseline demographic and clinical characteristics were comparable between groups. Mean age was similar in JW and non-JW patients (63.3 vs. 63.1 years), and women represented 30.8% and 32.1% of each group, respectively. EuroSCORE II values were also comparable (1.35 vs. 1.34), indicating similar low-risk surgical profiles.

Ten JW patients received preoperative iron supplementation or erythropoietin, whereas none of the non-JW patients underwent anemia optimization. Preoperative anemia, defined according to WHO criteria (hemoglobin < 13.0 g.dL^-1^ in males and < 12.0 g.dL^-1^ in females), was present in four JW patients (all male) and in 22 non-JW patients (15 males and 7 females). In some cases, particularly among non-JW patients, hemoglobin thresholds were not fully achieved; however, values were close to reference ranges, and surgical fitness was confirmed after multidisciplinary assessment considering clinical stability, urgency, and public healthcare constraints.

### Group matching

**Figure 3:**
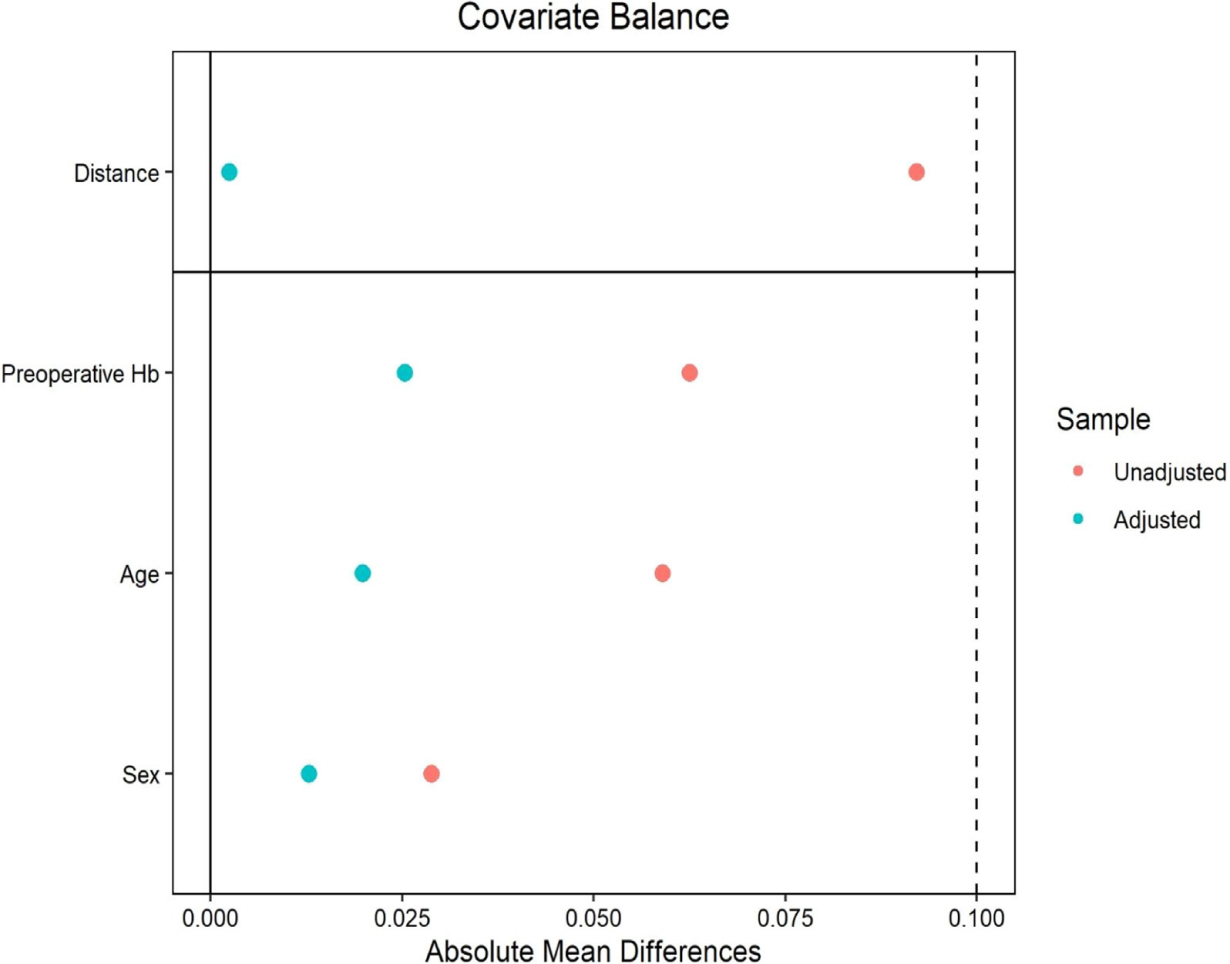
Balance between covariates before and after propensity score matching. Absolute Standardized Mean Differences (SMDs) are shown for each covariate comparing Jehovah’s Witnesses vs controls before matching (Unadjusted, red) and after matching (Adjusted, blue; 1:3 ratio). The vertical dashed line denotes the conventional balance threshold (|SMD| = 0.10), with values closer to 0 indicating better covariate balance. “Distance” denotes the propensity score (matching distance).

PSM matched all 26 Jehovah’s Witness patients to 78 controls. The median propensity score difference between each matched case and their respective controls was < 0.001 (maximum: < 0.001), indicating near-exact matching. Additionally, balance diagnostics revealed substantially improved standardized mean differences between groups after matching (distance: -0.002; age: 0.020; male sex: 0.013; preoperative hemoglobin: 0.025), whereas the corresponding pre-matching differences were larger (up to 0.092). These results are shown in Figure 3.

### Primary outcome: 30-day all-cause mortality

Regarding the primary outcome, no deaths occurred among JW patients within 30 days, whereas three deaths were observed in the non-JW group (Table 2). Estimates were imprecise due to low event counts.

### Secondary outcomes: major clinical events

**Figure 4:**
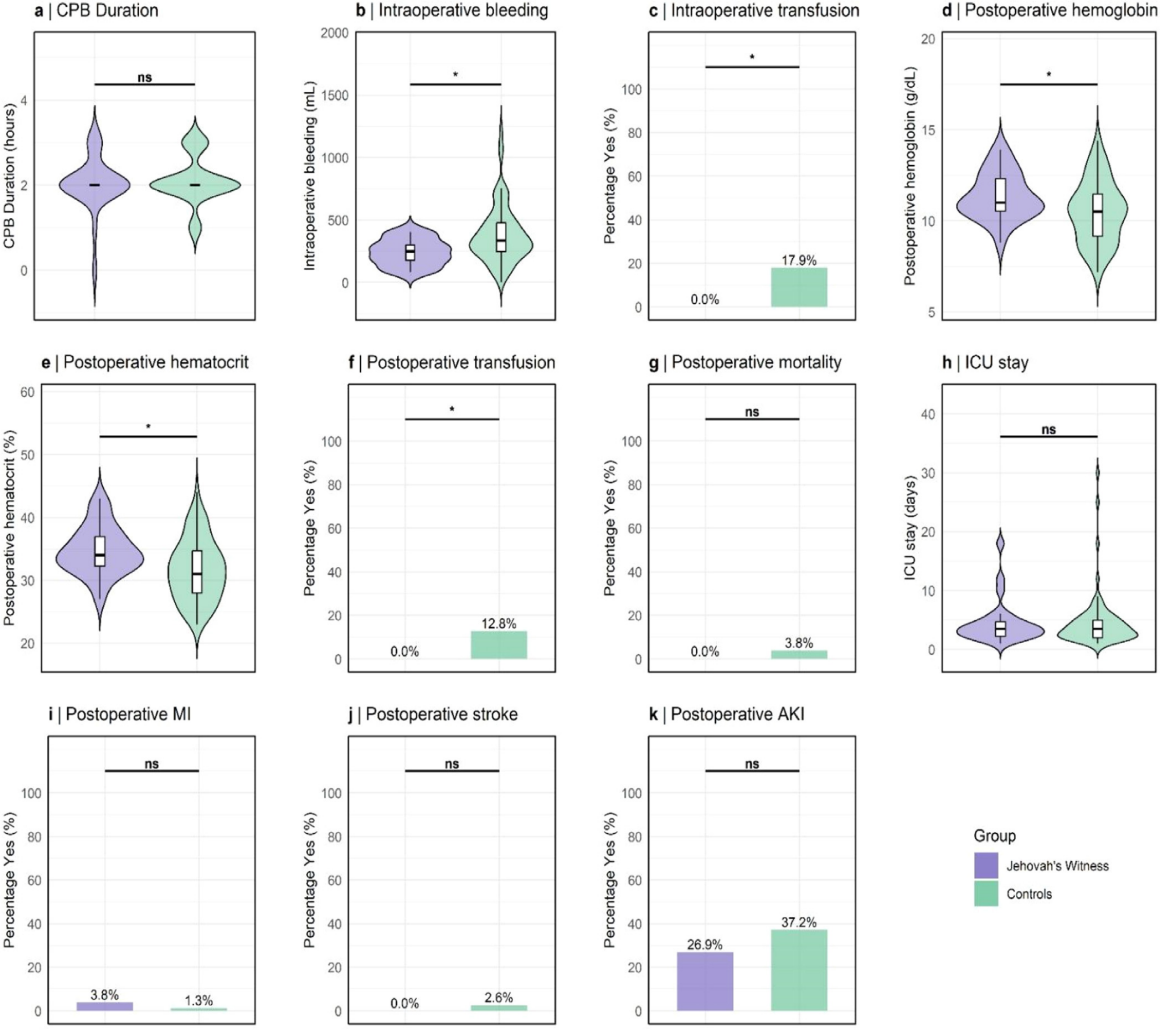
Comparison of intraoperative and postoperative variables between Jehovah’s Witnesses and matched controls. Continuous variables (a, b, d, e, and h) are shown as violin plots, while categorical variables (c, f, g, i, j, and k) are shown as bar plots. Statistically significant group differences based on logistic regression models are marked with an asterisk (*), and non-significant differences are labeled as “ns”. Jehovah’s Witnesses are represented in purple, controls, in green.

Within 30 days, none of the JW patients experienced stroke, and one MI (3.8%) was observed. AKI occurred in seven JW patients (26.9%). In the non-JW group, one MI (1.3%), two strokes (2.6%), and 29 cases of AKI (37.2%) were recorded (Table 2 and Fig. 4). Differences between groups for these outcomes were not statistically significant.

### Secondary outcomes: transfusion and perioperative blood management (Fig. 4)

None of the 26 JW patients required intraoperative or postoperative RBC transfusion. In contrast, 14 non-JW patients (17.9%) received intraoperative RBC transfusions (OR = 0.056; 95% CI 0‒0.707; p = 0.02) and 10 non-JW (12.8%) received postoperative RBC transfusions (OR = 0.101; 95% CI 0.0001‒0.884; p = 0.035), respectively; Table 2 and Fig. 2). Cell-saver use was universal among JW patients (26/26, 100%) and was available for 43 of 78 non-JW patients (55.1%).

### Secondary outcomes: hematologic and intraoperative variables (Fig. 4)

Preoperative hemoglobin levels were similar between groups (13.70 vs. 13.20 g.dL^-1^; p = 0.134). Postoperatively, JW patients had higher hemoglobin (11.37 vs. 10.49 g.dL^-1^; OR = 1.49; 95% CI 1.02–2.25; p = 0.035) and hematocrit levels (34.77% vs. 31.65%; OR = 1.18; 95% CI 1.04–1.37; p = 0.006). Cardiopulmonary bypass time was comparable between groups. Intraoperative blood loss was lower in JW patients (241 mL vs. 383 mL; OR = 0.55; 95% CI 0.27–0.99; p = 0.048; Table 2 and Fig. 2).

### Secondary outcomes: postoperative course (Fig. 4)

Duration of mechanical ventilation and intensive care unit length of stay were similar between groups (Table 2).

### Sensitivity analysis

Sensitivity analyses adjusting for additional clinical covariates (EuroSCORE II, ASA status, LVEF < 50%, diabetes, and hypertension; Table 1) yielded results consistent with the primary models, without meaningful changes in effect estimates (Supplementary Tables 1–5).

**Table 1:** Baseline characteristics and preoperative data of Jehovah’s Witnesses and matched Control patients.

Characteristics	Jehovah’s Witnesses (n = 26)	Controls (n = 78)	p-value
**Demographics**			
Age (yr)	63.4 (10.35)	63.2 (9.11)	0.647
Male sex, n (%)	18 (69.2)	53 (67.9)	0.903
**Clinical Status**			
EuroSCORE II	1.34 (0.87)	1.35 (1.40)	0.925
Preoperative hemoglobin (g.dL^-1^)	13.7 (1.01)	13.3 (1.58)	134
LVEF < 50%, n (%)	5 (19.2)	15 (19.2)	1
**ASA Physical Status, n (%)**			
II	0 (0.0)	11 (14.5)	0.387
III	19 (73.1)	44 (57.9)	
IV	7 (26.9)	21 (27.6)	
**Comorbidities, n (%)**			
Hypertension	22 (84.6)	70 (89.7)	0.478
Diabetes mellitus	14 (53.8)	41 (52.6)	0.910

Values are mean (SD) or number (proportion). p-values denote differences between groups using Student’s *t*-test or Mann-Whitney *U*-test for continuous variables and Chi-Square or Fisher’s exact test for categorical variables. ASA, American Society of Anesthesiologists; LVEF, Left Ventricular Ejection Fraction.

**Table 2:** Logistic regression results adjusted for matching variables (age, sex, preoperative hemoglobin) + EuroSCORE II.

Variable	Study Group: Jehovah's Witnesses (n = 26)	Study Group: Controls (n = 78)	OR (95% CI)	p-value
CPB duration (hours)^a^	2.04 (0.61)	2.11 (0.54)	0.610 (0.202–1.601)	0.287
Intraoperative bleeding (mL)^a^	241.76 (105.00)	383.39 (230.51)	0.557 (0.272–0.996)	0.048^b^
Intraoperative transfusion	0 (0.0%)	14 (17.9%)	0.081 (0.001–0.804)	0.028^b^
Postoperative death	0 (0.0%)	3 (3.8%)	0.427 (0.003–4.741)	0.543
Postoperative hb (g.dL^-1^)	11.37 (1.26)	10.49 (1.70)	1.491 (1.028–2.257)	0.035^b^
Postoperative hct (%)	34.77 (3.84)	31.65 (4.87)	1.189 (1.048–1.374)	0.006^c^
Postoperative ICU stay (days)^a^	4.50 (3.68)	4.59 (4.58)	1.160 (0.565–2.335)	0.679
Postoperative myocardial infarction	1 (3.8%)	1 (1.3%)	2.806 (0.208–37.974)	0.403
Postoperative renal dysfunction	7 (26.9%)	29 (37.2%)	0.625 (0.217–1.673)	0.355
Postoperative stroke	0 (0.0%)	2 (2.6%)	0.636 (0.004–9.738)	0.773
Postoperative transfusion	0 (0.0%)	10 (12.8%)	0.097 (0.001–0.850)	0.032^b^

Data: mean [SD] for continuous variables, n (%) for categorical. OR, Odds Ratio; CI, Confidence Interval. All models adjusted for matching variables (age, sex, preoperative hemoglobin) + EuroSCORE II.

a Log transformation applied when either group showed non-normality (Shapiro-Wilk p < 0.05). p-values are uncorrected.

b p < 0.05.

c p < 0.01.

## Discussion

This study describes perioperative management and short-term outcomes of JW patients undergoing CABG within a public tertiary healthcare system. Rather than providing definitive estimates of safety for rare events, our findings primarily offer an insight into the feasibility and implementation of structured PBM strategies in a transfusion-restricted population under routine public-hospital conditions.

The primary finding of this study concerns 30-day all-cause mortality, with no deaths observed among JW patients and no clear difference in estimated risk compared with non-JW patients, acknowledging the limited power to detect rare events. Beyond mortality, JW patients had lower exposure to RBC transfusion, a finding consistent with prior observational data and randomized evidence supporting restrictive transfusion strategies in cardiac surgery. Studies such as those by Chambault et al.^17^ and the TRICS III trial^9^ have demonstrated that restrictive transfusion approaches are not associated with worse clinical outcomes and may reduce transfusion-related risks. Within this framework, our results support the feasibility of CABG in transfusion-restricted patients while reinforcing the relevance of PBM principles, noting that these secondary findings remain exploratory. Furthermore, the well-established risks associated with allogeneic transfusion, including infection, immune modulation, and increased morbidity and mortality, support the rationale for restrictive transfusion strategies, as demonstrated in landmark trials by Carson et al.^8^ and Hébert et al.^18^

In addition, JW patients presented with higher postoperative hemoglobin levels and lower intraoperative blood loss compared with matched controls. These findings are consistent with structured blood conservation efforts and enhanced perioperative vigilance. As highlighted by Shander et al.,^19^ the JW population played a key role in the development of PBM principles, with implications extending beyond religious contexts.^20^ For patients who categorically refuse transfusion, strategies such as meticulous hemostasis, avoidance of unnecessary hemodilution, intraoperative cell salvage, and pharmacologic optimization are essential. When applied systematically, these principles may also benefit the broader CABG population, although their direct impact on hard clinical outcomes remains uncertain.

At our institution, surgical eligibility for JW patients did not rely on a fixed hemoglobin cutoff and decisions were individualized. We agree that higher preoperative hemoglobin levels are desirable and may offer greater physiological reserve in transfusion-restricted patients. However, optimal Hb targets depend on multiple patient- and procedure-related factors, including body size, surgical complexity, characteristics of extracorporeal circulation, and anticipated blood loss. Accordingly, Hb optimization in this cohort was individualized rather than protocol-driven, balancing these factors with clinical urgency and institutional constraints. Although Vaislic et al.^21^ proposed a preoperative Hb ≥ 14 g.dL^-1^, applying this threshold to our cohort would have excluded 42% of JW patients from surgery, despite the absence of clear differences in short-term outcomes compared with those with higher baseline Hb. This observation may suggest that rigid Hb criteria may unnecessarily delay or preclude surgical treatment without clear evidence of benefit.

Consistent with the individualized approach to preoperative hemoglobin optimization, perioperative transfusion decisions were not based on isolated numeric thresholds. Hemoglobin values were not used as absolute triggers, as acute blood loss with hemodynamic instability may precede measurable hematimetric decline. Likewise, hemodynamic criteria were not defined by rigid cutoffs for vasoactive drug dose, blood pressure, or cardiac output, given interindividual variability in physiologic tolerance. Point-of-care testing was routinely employed intraoperatively and postoperatively to assess hematologic and coagulation parameters when indicated; however, transfusion decisions ultimately relied on clinical judgment and multidisciplinary discussion, reflecting standard practice in cardiac surgery where timely intervention is essential.

This study has important limitations. Although PSM was applied, residual confounding cannot be excluded, particularly given the small JW cohort and limited overlap of some perioperative exposures. Differences in access to intraoperative blood conservation strategies also represent a key limitation: all JW patients had cell-saver availability, whereas only a subset of non-JW controls did, potentially favoring outcomes in the JW group and limiting causal interpretation. Stroke diagnosis was based on clinically triggered neuroimaging rather than systematic screening, and peri-procedural myocardial infarction was defined according to contemporary CABG-specific criteria. These factors may have led to under-ascertainment of asymptomatic events. The single-center, single-surgeon design reduces procedural variability but limits generalizability to other settings. Finally, multiple secondary and exploratory outcomes were analyzed without adjustment for multiplicity; given the limited sample size and imprecision for rare events, these findings should be interpreted as hypothesis-generating. Larger multicenter studies are needed to provide more precise estimates of the impact of blood management strategies on infrequent but clinically relevant outcomes.

Taken together, the observed findings suggest that structured preoperative preparation, meticulous intraoperative blood conservation, and restrictive transfusion practices may contribute to the generally similar short-term outcomes observed between JW and non-JW patients undergoing CABG. Rather than implying superiority or causality, these results highlight the potential value of applying PBM principles more broadly in contemporary cardiac surgery. Future multicenter, prospective studies are warranted to confirm these observations, better account for residual confounding, and further define the role of blood management strategies across diverse clinical and healthcare settings.

## Conclusion

In this retrospective observational study conducted in a public tertiary hospital, CABG in Jehovah’s Witness patients was feasible and associated with acceptable short-term outcomes. Despite system constraints, outcomes were generally comparable to those observed in non-JW patients, acknowledging the imprecision related to the small sample size. Rather than implying superiority, these findings highlight the relevance of structured PBM strategies in contemporary cardiac surgery. Further multicenter, prospective studies are warranted to confirm these observations and better define the role of PBM in diverse public healthcare settings.

## Declaration of generative AI and AI-assisted technologies in the manuscript preparation process

During the preparation of this work, the authors used ChatGPT (OpenAI, San Francisco, CA, USA) to assist with language editing, improvement of clarity and organization of the manuscript, and refinement of responses to peer reviewers. After using this tool, the authors carefully reviewed and edited all content as needed and take full responsibility for the accuracy and integrity of the final manuscript.

## Authors' contributions

Henrique Ryu Yamanaka Nakano: Conceptualization; methodology; data analysis; investigation; writing of the original draft and manuscript review & editing.

Guilherme Mota Filho: Data acquisition; investigation, and manuscript review & editing.

Bruno Camargo Calabria Araújo: data acquisition; investigation, and manuscript review & editing.

Heloísa Zogheib: Data acquisition; investigation, and manuscript review & editing.

Enzo Camargo Calabria Araújo: Data acquisition; investigation, and manuscript review & editing.

Leonardo Cardoso Saraiva: Data analysis and manuscript review & editing.

Jessica Silva Nicolau: Data acquisition; investigation, and manuscript review & editing.

Suely Pereira Zeferino: Data acquisition; investigation, and manuscript review & editing.

José Otávio Costa Auler Junior: Supervision; project administration, and critical revision of the manuscript.

## Data availability statement

The datasets generated and/or analyzed during the current study are available from the corresponding author upon reasonable request.

## Funding

The authors declare no funding support.

## Conflicts of interest

The authors declare that they have no conflicts of interest and that this study received no external funding.

## Appendix Supplementary materials

Image, application 1

## Footnotes

Supplementary material associated with this article can be found in the online version at doi:10.1016/j.bjane.2026.844765.
