# Supplementary material for: Coronary artery bypass grafting in Jehovah’s Witness patients: a retrospective propensity matched cohort study
Source: Braz J Anesthesiol. 2026 May 13;76(4):844765. doi: 10.1016/j.bjane.2026.844765 (PMC13285268; doi:10.1016/j.bjane.2026.844765)
Supplement: Supplementary file 1 [file mmc1.docx]

**BJAN-D-25-00601_ Supplementary Material**

**Supplementary Table 1** Logistic regression results adjusted for matching variables.

| **Variable** | **Study Group: Jehovah's Witnesses**  **(n = 26)** | **Study Group: Controls**  **(n = 78)** | **OR (95% CI)** | **p-value** |
| --- | --- | --- | --- | --- |
| CPB duration (hours)^a^ | 2.04 (0.61) | 2.11 (0.54) | 0.631 (0.200–1.617) | 0.313 |
| Intraoperative bleeding (mL)^a^ | 241.76 (105.00) | 383.39 (230.51) | 0.552 (0.270–0.989) | 0.046^b^ |
| Intraoperative transfusion | 0 (0.0%) | 14 (17.9%) | 0.056 (0.000–0.707) | 0.020^b^ |
| Postoperative death | 0 (0.0%) | 3 (3.8%) | 0.454 (0.003–5.085) | 0.575 |
| Postoperative Hb (g.dL^-1^) | 11.37 (1.26) | 10.49 (1.70) | 1.502 (1.033–2.280) | 0.032^b^ |
| Postoperative Hct (%) | 34.77 (3.84) | 31.65 (4.87) | 1.189 (1.049–1.374) | 0.006^c^ |
| Postoperative ICU stay (days)^a^ | 4.50 (3.68) | 4.59 (4.58) | 1.094 (0.520–2.243) | 0.807 |
| Postoperative myocardial infarction | 1 (3.8%) | 1 (1.3%) | 2.569 (0.180–35.897) | 0.453 |
| Postoperative renal dysfunction | 7 (26.9%) | 29 (37.2%) | 0.542 (0.176–1.523) | 0.251 |
| Postoperative stroke | 0 (0.0%) | 2 (2.6%) | 0.680 (0.005–10.712) | 0.808 |
| Postoperative transfusion | 0 (0.0%) | 10 (12.8%) | 0.101 (0.001–0.884) | 0.035^b^ |

Data: mean [SD] for continuous variables, n (%) for categorical. OR, Odds Ratio; CI, Confidence Interval. All models adjusted for matching variables (age, sex, preoperative hemoglobin).

^a^ Log transformation applied when either group showed non-normality (Shapiro-Wilk p < 0.05). p-values are uncorrected; ^b^ p < 0.05; ^c^ p < 0.01.

**Supplementary Table 2** Logistic regression results adjusted for matching variables + ASA status.

| **Variable** | **Study Group: Jehovah's Witnesses**  **(n = 26)** | **Study Group: Controls**  **(n = 78)** | **OR (95% CI)** | **p-value** |
| --- | --- | --- | --- | --- |
| CPB Duration (hours)^a^ | 2.04 (0.61) | 2.11 (0.54) | 0.644 (0.204–1.695) | 0.349 |
| Intraoperative Bleeding (mL)^a^ | 241.76 (105.00) | 383.39 (230.51) | 0.568 (0.273–1.027) | 0.061 |
| Intraoperative Transfusion | 0 (0.0%) | 14 (17.9%) | 0.096 (0.001–1.028) | 0.053 |
| Postoperative Death | 0 (0.0%) | 3 (3.8%) | 0.367 (0.003–4.128) | 0.468 |
| Postoperative Hb (g.dL^-1^) | 11.37 (1.26) | 10.49 (1.70) | 1.478 (1.011–2.256) | 0.044^b^ |
| Postoperative Hct (%) | 34.77 (3.84) | 31.65 (4.87) | 1.187 (1.044–1.375) | 0.008^c^ |
| Postoperative ICU Stay (Days)^a^ | 4.50 (3.68) | 4.59 (4.58) | 1.114 (0.545–2.236) | 0.762 |
| Postoperative Myocardial Infarction | 1 (3.8%) | 1 (1.3%) | 2.164 (0.159–30.130) | 0.531 |
| Postoperative Renal Dysfunction | 7 (26.9%) | 29 (37.2%) | 0.562 (0.188–1.541) | 0.268 |
| Postoperative Stroke | 0 (0.0%) | 2 (2.6%) | 0.551 (0.004–8.439) | 0.701 |
| Postoperative Transfusion | 0 (0.0%) | 10 (12.8%) | 0.069 (0.001–0.650) | 0.014^b^ |

Data: mean [SD] for continuous variables, n (%) for categorical. OR, Odds Ratio; CI, Confidence Interval. All models adjusted for matching variables (age, sex, preoperative hemoglobin) + ASA_status.

^a^ Log transformation applied when either group showed non-normality (Shapiro-Wilk p < 0.05). p-values are uncorrected; ^b^ p < 0.05; ^c^ p < 0.01.

**Supplementary Table 3** Logistic regression results adjusted for matching variables + diabetes.

| **Variable** | **Study Group: Jehovah's Witnesses**  **(n = 26)** | **Study Group: Controls**  **(n = 78)** | **OR (95% CI)** | **p-value** |
| --- | --- | --- | --- | --- |
| CPB Duration (hours)^a^ | 2.04 (0.61) | 2.11 (0.54) | 0.576 (0.188–1.528) | 0.244 |
| Intraoperative Bleeding (mL)^a^ | 241.76 (105.00) | 383.39 (230.51) | 0.553 (0.271–0.993) | 0.047^b^ |
| Intraoperative Transfusion | 0 (0.0%) | 14 (17.9%) | 0.077 (0.001–0.774) | 0.026^b^ |
| Postoperative Death | 0 (0.0%) | 3 (3.8%) | 0.426 (0.003–4.678) | 0.539 |
| Postoperative Hb (g.dL^-1^) | 11.37 (1.26) | 10.49 (1.70) | 1.502 (1.033–2.286) | 0.033^b^ |
| Postoperative Hct (%) | 34.77 (3.84) | 31.65 (4.87) | 1.193 (1.051–1.380) | 0.005^c^ |
| Postoperative ICU Stay (Days)^a^ | 4.50 (3.68) | 4.59 (4.58) | 1.160 (0.567–2.328) | 0.677 |
| Postoperative Myocardial Infarction | 1 (3.8%) | 1 (1.3%) | 2.781 (0.209–37.050) | 0.403 |
| Postoperative Renal Dysfunction | 7 (26.9%) | 29 (37.2%) | 0.620 (0.209–1.700) | 0.359 |
| Postoperative Stroke | 0 (0.0%) | 2 (2.6%) | 0.601 (0.004–9.494) | 0.746 |
| Postoperative Transfusion | 0 (0.0%) | 10 (12.8%) | 0.099 (0.001–0.859) | 0.033^b^ |

Data: mean [SD] for continuous variables, n (%) for categorical. OR, Odds Ratio; CI, Confidence Interval. All models adjusted for matching variables (age, sex, preoperative hemoglobin) + Diabetes.

^a^ Log transformation applied when either group showed non-normality (Shapiro-Wilk p < 0.05). p-values are uncorrected; ^b^ p < 0.05; ^c^ p < 0.01.

**Supplementary Table 4** Logistic regression results adjusted for matching variables + hypertension.

| **Variable** | **Study Group: Jehovah's Witnesses**  **(n = 26)** | **Study Group: Controls**  **(n = 78)** | **OR (95% CI)** | **p-value** |
| --- | --- | --- | --- | --- |
| CPB Duration (hours)^a^ | 2.04 (0.61) | 2.11 (0.54) | 0.585 (0.187–1.535) | 0.252 |
| Intraoperative Bleeding (mL)^a^ | 241.76 (105.00) | 383.39 (230.51) | 0.555 (0.268–0.998) | 0.049^b^ |
| Intraoperative Transfusion | 0 (0.0%) | 14 (17.9%) | 0.086 (0.001–0.854) | 0.033^b^ |
| Postoperative Death | 0 (0.0%) | 3 (3.8%) | 0.341 (0.002–3.926) | 0.437 |
| Postoperative Hb (g.dL^-1^) | 11.37 (1.26) | 10.49 (1.70) | 1.602 (1.088–2.479) | 0.016^b^ |
| Postoperative Hct (%) | 34.77 (3.84) | 31.65 (4.87) | 1.211 (1.063–1.407) | 0.003^c^ |
| Postoperative ICU Stay (Days)^a^ | 4.50 (3.68) | 4.59 (4.58) | 1.153 (0.560–2.326) | 0.692 |
| Postoperative Myocardial Infarction | 1 (3.8%) | 1 (1.3%) | 3.017 (0.222–41.177) | 0.373 |
| Postoperative Renal Dysfunction | 7 (26.9%) | 29 (37.2%) | 0.610 (0.210–1.635) | 0.331 |
| Postoperative Stroke | 0 (0.0%) | 2 (2.6%) | 0.626 (0.004–9.690) | 0.765 |
| Postoperative Transfusion | 0 (0.0%) | 10 (12.8%) | 0.085 (0.001–0.740) | 0.021^b^ |

Data: mean [SD] for continuous variables, n (%) for categorical. OR, Odds Ratio; CI Confidence Interval. All models adjusted for matching variables (age, sex, preoperative hemoglobin) + Hypertension.

^a^ Log transformation applied when either group showed non-normality (Shapiro-Wilk p < 0.05). p-values are uncorrected; ^b^ p < 0.05; ^c^ p < 0.01.

**Supplementary Table 5** Logistic regression results adjusted for matching variables + heart failure.

| **Variable** | **Study Group: Jehovah's Witnesses**  **(n = 26)** | **Study Group: Controls**  **(n = 78)** | **OR (95% CI)** | **p-value** |
| --- | --- | --- | --- | --- |
| CPB Duration (hours)^a^ | 2.04 (0.61) | 2.11 (0.54) | 0.628 (0.206‒1.629) | 0.315 |
| Intraoperative Bleeding (mL)^a^ | 241.76 (105.00) | 383.39 (230.51) | 0.554 (0.272‒0.99) | 0.046^b^ |
| Intraoperative Transfusion | 0 (0.0%) | 14 (17.9%) | 0.083 (0.001‒0.806) | 0.028^b^ |
| Postoperative Death | 0 (0.0%) | 3 (3.8%) | 0.436 (0.003‒4.891) | 0.555 |
| Postoperative Hb (g.dL^-1^) | 11.37 (1.26) | 10.49 (1.70) | 1.485 (1.024‒2.247) | 0.036^b^ |
| Postoperative Hct (%) | 34.77 (3.84) | 31.65 (4.87) | 1.187 (1.047‒1.371) | 0.006^c^ |
| Postoperative ICU Stay (Days)^a^ | 4.50 (3.68) | 4.59 (4.58) | 1.131 (0.539‒2.324) | 0.74 |
| Postoperative Myocardial Infarction | 1 (3.8%) | 1 (1.3%) | 2.644 (0.184‒36.033) | 0.438 |
| Postoperative Renal Dysfunction | 7 (26.9%) | 29 (37.2%) | 0.614 (0.212‒1.645) | 0.338 |
| Postoperative Stroke | 0 (0.0%) | 2 (2.6%) | 0.646 (0.004‒9.973 | 0.781 |
| Postoperative Transfusion | 0 (0.0%) | 10 (12.8%) | 0.098 (0.001‒0.862) | 0.033^b^ |

Data: mean [SD] for continuous variables, n (%) for categorical. OR, Odds Ratio; CI, Confidence Interval. All models adjusted for matching variables (age, sex, preoperative hemoglobin) + Heart_Failure_EF_lt_50.

^a^ Log transformation applied when either group showed non-normality (Shapiro-Wilk p < 0.05). p-values are uncorrected; ^b^ p < 0.05; ^c^ p < 0.01.
